# Expanding Youth-Friendly HIV Self-Testing Services During the COVID-19 Pandemic: Qualitative Analysis of a Crowdsourcing Open Call in Nigeria

**DOI:** 10.2196/46945

**Published:** 2024-04-30

**Authors:** Onyekachukwu Anikamadu, Oliver Ezechi, Alexis Engelhart, Ucheoma Nwaozuru, Chisom Obiezu-Umeh, Ponmile Ogunjemite, Babatunde Ismail Bale, Daniel Nwachukwu, Titilola Gbaja-biamila, David Oladele, Adesola Z Musa, Stacey Mason, Temitope Ojo, Joseph Tucker, Juliet Iwelunmor

**Affiliations:** 1 Brown School of Social Work Washington University in St. Louis St Louis, MO United States; 2 Clinical Sciences Department Nigerian Institute of Medical Research Lagos Nigeria; 3 Division of Infectious Disease Washington University School of Medicine in St. Louis St Louis, MO United States; 4 Department of Implementation Science Wake Forest School of Medicine Winston-Salem, NC United States; 5 Department of Behavioral Science and Health Education College for Public Health and Social Justice Saint Louis University St Louis, MO United States; 6 Department of Human Anatomy Federal University of Technology Akure Nigeria; 7 Department of Optometry University of Benin Benin City Nigeria; 8 Division of Infectious Diseases School of Medicine University of North Carolina at Chapel Hill Chapel Hill, NC United States; 9 Faculty of Infectious and Tropical Diseases London School of Hygiene and Tropical Medicine London United Kingdom

**Keywords:** crowdsourcing, World AIDS Day, HIV, self-testing, young people, COVID-19 pandemic restrictions, Nigeria, HIV self-testing, health promotion, crowdsourcing open call, young adult

## Abstract

**Background:**

HIV self-testing (HIVST) among young people is an effective approach to enhance the uptake of HIV testing recommended by the World Health Organization. However, the COVID-19 pandemic disrupted conventional facility-based HIV testing services, necessitating the exploration of innovative strategies for the effective delivery of HIVST.

**Objective:**

This study analyzed the outcomes of a digital World AIDS Day crowdsourcing open call, designed to elicit youth responses on innovative approaches to promote HIVST among young people (14-24 years) in Nigeria during COVID-19 restrictions.

**Methods:**

From November 2 to 22, 2020, a World AIDS Day 2020 crowdsourcing open call was held digitally due to COVID-19 restrictions. The crowdsourcing open call followed World Health Organization standardized steps, providing a structured framework for participant engagement. Young people in Nigeria, aged 10-24 years, participated by submitting ideas digitally through Google Forms or email in response to this crowdsourcing open call prompt: “How will you promote HIV self-testing among young people during COVID-19 pandemic?” Data and responses from each submission were analyzed, and proposed ideas were closely examined to identify common themes. Four independent reviewers (AE, SM, AZM, and TG) judged each submission based on the desirability, feasibility, and impact on a 9-point scale (3-9, with 3 being the lowest and 9 being the highest).

**Results:**

The crowdsourcing open call received 125 eligible entries, 44 from women and 65 from men. The median age of participants was 20 (IQR 24-20) years, with the majority having completed their highest level of education at the senior secondary school level. The majority of participants lived in the South-West region (n=61) and Lagos state (n=36). Of the 125 eligible entries, the top 20 submissions received an average total score of 7.5 (SD 2.73) or above. The panel of judges ultimately selected 3 finalists to receive a monetary award. Three prominent themes were identified from the 125 crowdsourcing open call submissions as specific ways that HIVST can adapt during the COVID-19 pandemic: (1) digital approaches (such as gamification, photoverification system, and digital media) to generate demand for HIVST and avoid risks associated with attending clinics, (2) awareness and sensitization through existing infrastructures (such as churches, schools, and health facilities), and (3) partnerships with influencers, role models, and leaders (such as religious and youth leaders and social influencers in businesses, churches, organizations, and schools) to build trust in HIVST services.

**Conclusions:**

The crowdsourcing open call effectively engaged a diverse number of young people who proposed a variety of ways to improve the uptake of HIVST during the COVID-19 pandemic. Findings contribute to the need for innovative HIVST strategies that close critical knowledge and practice gaps on ways to reach young people with HIVST during and beyond the pandemic.

**Trial Registration:**

ClinicalTrials.gov NCT04710784; https://clinicaltrials.gov/study/NCT04710784

## Introduction

Nigeria stands as the fourth-highest contributor to the global burden of HIV and has the largest HIV burden in sub-Saharan Africa [1,2]. An estimated 1.9 million Nigerians were living with HIV in 2018, setting the national HIV prevalence among those aged 15 to 49 years at 1.5% [3]. Since then, as of 2020, rates have remained constant, with only 67% of those affected aware of their status and only 53% receiving treatment [3]. Even with strengthened interventions over the past few decades, Nigeria still has a high burden of HIV, and a high number of young people (14-24 years) acquire the virus [3].

HIV self-testing (HIVST) is an important step that can enhance the uptake of essential HIV prevention services and is recommended by the World Health Organization [4]. HIVST is an antibody test that allows individuals to conveniently test themselves at home or in a private setting, yielding results within 20 minutes [5]. Recognized as a safe and effective method for increasing testing rates, especially among young people, HIVST has long been considered an alternative to clinical testing [4]. With the ability to collect their own samples and conduct the test at their own convenience, individuals gain autonomy in the testing process [4]. Although HIVST had been gaining traction prior to the COVID-19 pandemic, COVID-19 regulations hampered HIV testing, hastening the creation and scale-up of HIVST services [6].

The COVID-19 pandemic significantly impacted and disturbed the global health care system to varying degrees, with HIV prevention and treatment programs among those that were not spared [7]. A shift in the health system’s focus impacted all health programs not only in Nigeria but around the world. Many resources were redirected due to the COVID-19 pandemic, causing disruptions in existing clinical and community-based services surrounding prevention, treatment, and curative services, including those of HIV [8,9]. HIVST can play a critical role in decentralizing services, guaranteeing the use of HIV testing services during periods of uncertainties. It allows both beneficiaries and health care providers to adhere to physical distancing guidelines and limits the danger of exposure to transmission. While certain populations of Nigerian young people are at high risk of being infected with HIV, there is minimal evidence on measures to improve HIVST uptake among young people in Nigeria during the COVID-19 pandemic. An understanding of the strategies would be valuable to Nigerian young people and policy makers as well as global researchers implementing interventions in Nigeria or those who desire to adapt interventions to different contexts, particularly during disruptions such as those caused by the pandemic. To address this gap, we launched a digital crowdsourcing open call to elicit ideas for promoting HIVST during the pandemic. Crowdsourcing is an open innovation approach, often used as a tool to gather ideas, innovations, or information for specific purposes [10]. It is an effective strategy for communicating and designing interventions related to HIV and sexual health [11,12]. The goal of this qualitative study was to uncover recurring themes in a digital World AIDS Day (WAD) crowdsourcing open call for youth responses on how to increase HIVST among Nigerian young people during the COVID-19 pandemic.

## Methods

### Overview

**Figure 1:**
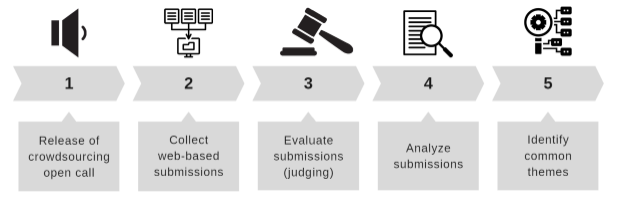
Five-step methodology.

Our crowdsourcing open call consisted of a 5-step process including digital crowdsourcing open call, web-based submissions, judging, analysis of themes, and common themes identified (Figure 1) throughout the design and implementation, data collection, and data analysis phases.

### Digital Crowdsourcing Open Call Design and Implementation

For this study, we used crowdsourcing, which involves a group of individuals working together to solve a problem and then publicly share the solutions [13,14]. The 4 Youth By Youth (4YBY) team announced a crowdsourcing open call to promote HIVST among young people in Nigeria from November 2 to 22, 2020. 4YBY is a team of young people, health professionals, activists, and entrepreneurs from diverse backgrounds, who are united by the shared passion to advance Nigerian youth participation in creating innovative, sustainable HIV prevention services. The “World’s AIDS Day HIV Self-Testing Contest” was held on December 1, 2020, to commemorate the annual WAD celebration. Due to the COVID-19 pandemic, we only used web-based engagement to recruit Nigerian young people between the ages of 14 and 24 years to take part in the crowdsourcing open call. To guarantee that a varied range of Nigerian young people from various backgrounds were involved, purposive sampling procedures were used [15]. The 4YBY team sent the crowdsourcing open call information to youth listservs and advertised on all social media platforms, including Instagram, Facebook, WhatsApp, and a website. The goal of the digital crowdsourcing open call was to provide open and safe areas for Nigerian young people to express their thoughts and ideas about how to promote HIVST during the pandemic. Individual demographic and contact information such as name, number, age, gender identity, education, and location were collected for each entrant.

### Web-Based Collection of Submission Data

Participants responded to the prompt “How might you promote HIV self-testing among young people during COVID-19 measures?” using either Google Forms or email. Each submission had a word limit of 150 words to succinctly capture their unique ideas for HIV promotion throughout the pandemic. Consent to participate and demographic information were also obtained from each participant. The requested demographic information included contact information, age, gender, relationship status, current location, highest level of completed education, and occupation. Individuals had the option of not reporting sociodemographic characteristics.

### Judging

Two research members (CO-U and UN) checked for the eligibility of each submission. Four independent reviewers (AE, SM, AZM, and TG) rated participant entries based on defined judging criteria, which included desirability, feasibility, and impact (Textbox 1). Each submission received either a 1 (low), 2 (moderate), or 3 (high) for each of the 3 defined judging criteria, with the highest overall combined score being 9. This process was adapted from the human-centered, design thinking framework [15,16]. The first, second, and third place contestants each earned a monetary prize for their outstanding submission: 50,000 Naira (approximately US $139) for third place, 150,000 Naira (approximately US $417) for second place, and 250,000 Naira (approximately US $694) for third place.

**4 Youth By Youth World AIDS Day crowdsourcing open call judging criteria (based on a 9-point scale: 3-9, with 3 as the lowest and 9 as the highest).**

Desirability (1-3): Concept is appealing to young people and is affordable, accessible, and confidential.

Feasibility (1-3): Concept is practical in terms of implementation and resource availability.

Impact (1-3): Concept has the ability to influence young people to self-test for HIV and can reach young people in Nigeria.

### Data Analysis

Following the crowdsourcing open call’s conclusion, staff deidentified each entry and used summary statistics to compile the participants’ demographic data. A thematic analysis was conducted using open coding that assigns themes to capture specific ideas and axial coding, which explores linkages between concepts and categories and determines common themes [17,18]. The coding exercises were completed in a Google Spreadsheet by trained staff who were not part of the study team, and then a thematic codebook was constructed and used to assess qualitative data. Each of the 2 staff members (OA and AE) coded the crowdsourcing open call entries separately before meeting to resolve any discrepancies in coding judgments. The codebook was then created by consensus between the 2 researchers (CO-U and UN) [18]. Throughout the coding process, recurring themes were identified, and codes were created to meet the HIVST promotion themes. The staff members reread each submission a final time after developing the initial codebook to fine-tune the final codebook. Snapshots of entries that fit in each code were included in the final codebook. This was then forwarded to a third party for final examination and approval.

### Ethical Considerations

Regulatory approval to conduct the research was received from the Nigerian Institute of Medical Research Institutional Review Board (Project #: IRB 18/028). Informed consent was obtained from all participants involved in the study. Following the conclusion of the crowdsourcing open call, each entry was deidentified to ensure anonymity. These measures were implemented to uphold ethical standards, ensuring participant protection and transparency throughout the research process. Additionally, monetary compensation was awarded to first-, second-, and third-place submissions.

## Results

### Characteristics of Entrant Submissions

The 2020 digital WAD crowdsourcing open call received a total of 153 entries from Nigerians aged 14 to 24 years, of which 83.7% (n=128) were through Google Forms, and 16.3% (n=25) were by email. Of the 153 entries, there were 125 unique submissions identified after duplicates (n=24), and those submissions that could not be scored based on our inclusion criteria (n=4) were removed. Characteristics and demographics were collected for the 125 eligible, unique submissions.

The majority of the entrants were male (n=65, 52%). There were 44 (35.2%) female individuals, 1 (0.8%) individual preferred not to say their gender, and 15 (12%) gender responses were left missing. The average age of the entrants was 19.96 (SD 2.73) years, and the median age was also 20 (IQR 24-20) years. The breakdown of the number of individuals per age group is as follows: 48 (38.4%) individuals aged 14-19 years and 64 (51.2%) individuals aged 20-24 years. The highest level of education obtained by participants was mostly senior secondary school (n=61, 48.8%), followed by some tertiary school (n=22, 17.6%), bachelor’s degree (n=17, 13.6%), junior secondary school (n=4, 3.2%), primary school (n=2, 1.6%), and master’s degree (n=2, 1.6%). In total, 15 (12%) individuals did not report their highest level of education. The majority of entrants lived in Lagos state (n=36, 28.8%) and in the South-West region (n=61, 48.8%). Table 1 includes the characteristics of the eligible submissions.

Of the 125 eligible submissions, the mean score of the submissions was 5.42 (1.65). In total, 20 participants were selected as finalists with their submissions scoring as 7.5 or above. Three submissions were selected as the top submissions and were given a prize (Multimedia Appendix 1).

The average word count of Google Forms submissions was 425.27 (SD 392.91) words. Two videos were submitted with lengths of 4.3 and 6.4 minutes. A total of 5 submissions included images, 1 submission consisted of a video and images, 1 entrant submitted a PowerPoint (Microsoft Corp) presentation file, and 4 submissions included a combination of images and text. All other entries were text submissions.

**Table 1:** Characteristics of submissions (N=125 eligible submissions).

Characteristics of submissions		Values
Google Forms, n (%)		100 (80)
Email, n (%)		25 (20)
**Sex, n (%)**		
	Female	44 (35.2)
	Male	65 (52)
	Preferred not to say	1 (0.8)
	Missing	15 (12)
**Age**		
	14-19 years, n (%)	48 (38.4)
	20-24 years, n (%)	64 (51.2)
	Missing, n (%)	13 (10.4)
	Mean (SD)	19.96 (2.73)
	Median (IQR)	20 (24-20)
**Education, n (%)**		
	Primary school	2 (1.6)
	Junior secondary school	4 (3.2)
	Senior secondary school	61 (48.8)
	Some tertiary school	22 (17.6)
	Bachelor’s degree	17 (13.6)
	Master’s degree	2 (1.6)
	Missing	15 (12)
**Location (by state), n (%)**		
	Abia	1 (0.8)
	Abuja	4 (3.2)
	Akure	1 (0.8)
	Akwa Ibom	1 (0.8)
	Anambra	2 (1.6)
	Bauchi	1 (0.8)
	Bayelsa	1 (0.8)
	Benue	3 (2.4)
	Cross River	6 (4.8)
	Delta	1 (0.8)
	Ebonyi	1 (0.8)
	Ekiti	2 (1.6)
	Enugu	5 (4)
	Imo	2 (1.6)
	Kaduna	6 (4.8)
	Kano	1 (0.8)
	Kogi	2 (1.6)
	Kwara	2 (1.6)
	Lagos	36 (28.8)
	Nasarawa	1 (0.8)
	Ogun	6 (4.8)
	Ondo	3 (2.4)
	Osun	3 (2.4)
	Oyo	10 (8)
	Plateau	2 (1.6)
	Rivers	6 (4.8)
	Yobe	2 (1.6)
	Missing	14 (11.2)
**Location (by region), n (%)**		
	Central	4 (3.2)
	East-Central	2 (1.6)
	North-Central	10 (8)
	North-East	3 (2.4)
	North-West	7 (5.6)
	South-East	21 (16.8)
	South-South	3 (2.4)
	South-West	61 (48.8)
	Missing	14 (11.2)
**Score, mean (SD)**		5.42 (1.65)
	1-3.5	28 (22.4)
	4-6.5	68 (54.4)
	7-9	30 (24.0)
	Above 7.5 (semifinalists)	20 (16.0)

### Analysis of Themes

#### Overview

After analyzing all 125 eligible submissions, 3 key themes were identified from the crowdsourcing open call submissions as specific ways that HIVST can adapt to the COVID-19 pandemic (theme 1) and the possibilities as well as facilitators of HIVST during the COVID-19 pandemic (themes 2 and 3). Common strategies to promote HIVST during and after COVID-19 were through (1) digital approaches (such as gamification, photoverification system, and digital media) to generate demand for HIVST and avoid risks associated with attending clinics, (2) awareness and sensitization through existing infrastructures (such as churches, schools, and health facilities), and (3) partnerships with influencers, role models, and leaders (such as religious and youth leaders and social influencers in businesses, churches, organizations, and schools) to build trust in HIVST services (Multimedia Appendix 2).

#### Theme 1: Digital Approaches to Generate Demand for HIVST and Avoid Risks Associated With Attending Clinics

**Figure 2:**
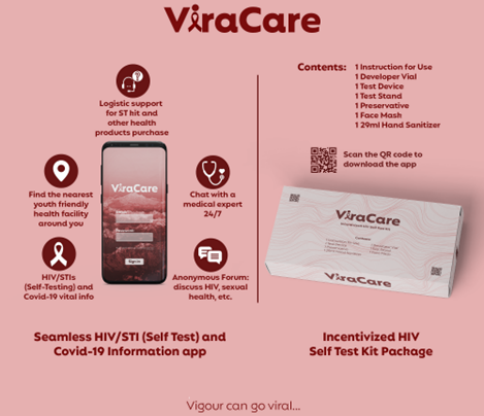
ViraCare, an example of entrants’ proposed mobile apps that provide knowledge on HIV self-testing and COVID-19 testing as well as linkage to testing facilities. STI: sexually transmitted infection.

This was a recurring theme that appeared to be desirable, feasible, and impactful to young people in Nigeria. Because COVID-19 can be spread through airborne transmission and social distancing regulations were put into place, young people saw digital media as a safe method to transmit awareness and information regarding HIV and HIVST. Young people established ideas to educate and promote HIVST through digital media engagement avenues such as Facebook, Twitter, Instagram, WhatsApp, gamification, and mobile apps. For example, entrants proposed creating mobile apps that provide knowledge on HIVST and COVID-19 testing as well as linkage to testing facilities (Figure 2). The top finalist suggested an interactive health gaming app called “Bambam” that allows users to access and monitor their health and HIV status coupled with competitive incentives to keep users engaged and encourage peer synergy. Because many young people in Nigeria have access to the internet and digital devices, participants mentioned the importance of using digital approaches as a medium for HIV and HIVST awareness campaigns to limit social gatherings and limit the influx of people to high-traffic areas such as hospitals. Submission examples are presented in Multimedia Appendix 2.

#### Theme 2: Awareness and Sensitization Through Existing Infrastructures

This was a common theme that arose even during the COVID-19 pandemic. Many submissions suggested that existing facilities and infrastructures can still be used as resources for HIV information and as testing sites. They emphasized the importance of wearing face masks, applying hand sanitizer, and social distancing while at these places. A few participants mentioned the idea of creating awareness through religious gatherings, as churches and religious bodies were still allowed to be open during the pandemic. Other participants made it clear that holding discussions and seminars in physical locations gives rise to more social and personal connections. However, participants mentioned it is important to avoid large gatherings. Submission examples are presented in Multimedia Appendix 2.

#### Theme 3: Partnerships With Influencers, Role Models, and Leaders to Build Trust in HIVST Testing Services

**Figure 3:**
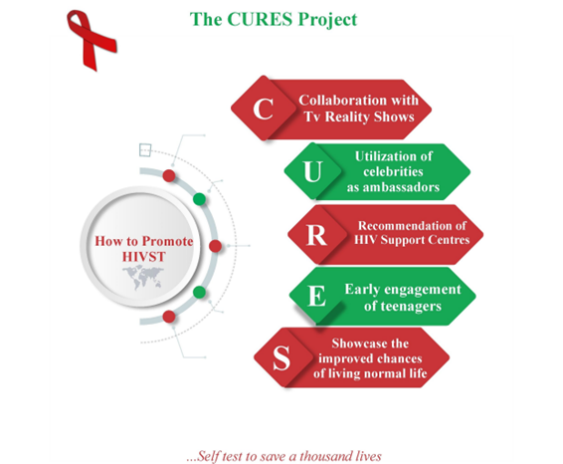
CURES, a participant submission that explained the use of 5 major tools to promote HIVST among young people. HIVST: HIV self-testing.

This was portrayed as a strategy that would impact young people in Nigeria and influence them to self-test for HIV. Participants mentioned collaborating with people such as social media influencers, religious and community leaders, and physicians to educate and sensitize young people in Nigeria on HIV and link them to self-testing kits and testing services. Participants suggested using celebrities as ambassadors to promote and encourage HIVST among young people (Figure 3). Entrants also proposed the idea of leaders reaching out, through trained personnel and volunteers, to individuals in rural and more local areas, as these individuals may not have access to internet services. Submission examples are presented in Multimedia Appendix 2.

Additionally, a few participants also mentioned similarities between the fight against COVID-19 with that of HIV. Participants recognized the comparability between COVID-19 and HIV and how HIV facility-based testing is stalled due to COVID-19 regulations such as facilities shutting down or social distancing guidelines being initiated. Participants highlighted the accessibility of HIVST, in which self-testing for HIV can be administered in an individual’s own home and privacy.

### Follow-Up Activities

Following the conclusion of the digital crowdsourcing event, the top-scored ideas generated informed the refinement of a youth-friendly HIVST intervention delivery, particularly focusing on enhancing linkage to care. This refined intervention is to be evaluated in a randomized controlled trial (ClinicalTrials.gov NCT04710784).

## Discussion

### Principal Findings

This digital WAD crowdsourcing open call administered during the COVID-19 pandemic generated ideas on how HIVST uptake can be increased among Nigerians in the course of the COVID-19 pandemic. The study illustrated efforts to reach young people creatively to facilitate HIVST while being compliant with the COVID-19 pandemic. Our findings also highlight accessible, affordable, and feasible ways to create awareness of HIVST throughout the COVID-19 pandemic and after the pandemic.

Our data suggest that HIVST can be effectively used to meet the demands of young people’s HIV testing during the pandemic. The COVID-19 pandemic created countless barriers that required the public to rethink a way around the norm and develop new strategies to overcome the obstacles, specifically reconsidering how to promote and fulfill HIVST services [19]. For example, across Nigeria, there were many disruptions to health care services [20], including those of HIV and sexually transmitted infections [9,21]. Yet, there was a need for innovative public health and social measures to reduce the disruptions caused by the pandemic [22]. Our crowdsourcing open call illustrated how a digital program can be used as a strategy to promote HIVST services that are compliant with the COVID-19 guidelines carried out in Nigeria [23]. Digital activities such as our crowdsourcing open call allowed for COVID-19 mitigation measures to continue to occur successfully while being compliant with government guidance [24,25].

Participants generated ideas on how HIVST distribution can be modified to adhere to the ongoing restrictions caused by the pandemic through channels that are digital as well as existing infrastructures and partnerships with key players. The ideas proposed can help form future campaigns, specifically during a time of limited physical interaction, and can pave the way toward more innovative and cost-saving techniques.

This study was held completely digitally due to the COVID-19 pandemic. It was a safe way to connect with individuals across Nigeria and administer new ideas that followed COVID-19 regulations. Although a large number of individuals participated in our crowdsourcing open call, it was significantly less than our crowdsourcing open calls in previous years 2018 and 2019 [15]. This could be related to competing COVID-19 demands. Though we gave the options of both web-based or offline submissions, all submissions were received via email or Google Forms. We recognize that technological advancements are continually occurring globally, and many young people have access to a mobile device, laptop, or computer [26]. In Nigeria, 63.8% of the population had internet access in 2020 [27]. However, internet connections are not always reliable and lead to issues during web-based learning or streaming [26]. Because our digital crowdsourcing open call was held via Zoom (Zoom Video Communications), this may have averted individuals from the crowdsourcing open call, knowing that they may have a possibility of internet connection issues.

However, as in previous years, the crowdsourcing open call engaged a wide range of young people and allowed them to voice their ideas on how to promote HIVST through various innovative strategies [15]. Crowdsourcing is not only a strategic way to receive solutions [9,10] but also very unique in the way that it includes individuals with different perspectives and creates meaningful participation among young people [13,14,28]. Our crowdsourcing open call accentuated this with youth participation and the development of ideas to prospectively scale up HIVST among young people and in communities throughout Nigeria. Harnessing young people’s concepts through crowdsourcing open calls captivates young people’s perspectives and potentially enhances mobilization efforts. Young people generated adapted, effective, and original strategies relating to the design, distribution, and education of HIVST kits during the COVID-19 era. We showed how young people can engage, produce innovative approaches, and create in-person or web-based connections.

### Limitations

This study has several limitations. First, most ineligible entries were those submitted via Google Forms, with 24 as duplicates and 2 as submissions that could not be scored. Second, many submissions included text that did not specifically answer the crowdsourcing open-call question, such as those that defined HIV and those that described the burden of HIV in Nigeria. These limitations highlight a need for better communication and marketing of the crowdsourcing event so individuals fully understand the objective of the crowdsourcing open call and prompt being asked. Third, this study inevitably had selection bias, in which some individuals may have been more likely selected than others, and the group of participants may not have been equally representative of all ages of young people in Nigeria (14 to 24 years). In addition, the crowdsourcing open call was completely web-based, and submissions were submitted through Google Forms and email, which may have excluded individuals who wanted to participate but had limited or no access to the internet. Fourth, analytical bias may have been introduced during the evaluation or scoring process. Finally, demographic information was more frequently missing in email submissions (ie, age and gender), whereas participants who submitted via Google Forms filled in the age and gender question.

Findings from this study will help inform and improve strategies of HIVST to increase HIVST uptake in Nigeria during the COVID-19 pandemic and for years to come in the postpandemic era. This study implies that crowdsourcing and involvement of young people are valuable in identifying current and unconventional HIVST uptake strategies, particularly during an ongoing pandemic, and should also be considered in other aspects of research.

### Conclusions

Though the study has many limitations, the crowdsourcing open call engaged a large, diverse number of young people through digital connections. The entrants suggested a diverse range of innovative techniques to increase the uptake of HIVST for vulnerable young people in Nigeria. Their tailored strategies to promote HIVST during the COVID-19 pandemic indicate that HIVST is a feasible method of testing despite barriers relating to physical interactions and in-person testing facilities. Findings from our crowdsourcing open call will inform future research on promoting HIVST and can contribute to the sustainability of HIVST even in times of unprecedented crises.

## Appendix

Multimedia Appendix 1 Top 3 submissions identified.

Multimedia Appendix 2 Emerging themes and examples of submissions.

## Abbreviations

4YBY: 4 Youth By Youth
HIVST: HIV self-testing
WAD: World AIDS Day

## Data Availability

The data generated and analyzed in this study are not publicly available due to privacy and confidentiality considerations but are available from the corresponding author on reasonable request.
