# Supplementary material for: Expanding Youth-Friendly HIV Self-Testing Services During the COVID-19 Pandemic: Qualitative Analysis of a Crowdsourcing Open Call in Nigeria
Source: JMIR Form Res. 2024 Apr 30;8:e46945. doi: 10.2196/46945 (PMC11094596; doi:10.2196/46945)
Supplement: Multimedia Appendix 1 [file formative_v8i1e46945_app1.docx]

| **Top Three Submissions** | **Quotes** |
| --- | --- |
|  |  |
| 1 - 1st place  (Score=7) | “Bambam is an interactive health app for youths to access their health status especially reproductive and sexual status and most importantly monitor their HIV status. Using bambam comes with lots of value added advantages, such as free health bies including free medical consultancy and tests, and free vouchers and gift items. Points can be gained and acquired on bambam and points converted to freebies. BamBam Game has a leaderboard rating system to encourage competition between peers.”  “**Why Bambam? To effectively and efficiently mobilize youths in HIV awareness an enabling value-based community is of utmost importance. This is the community we are proposing through bambam.**(#e010, female, 22 yrs) |
| 2 - 2nd place  (Score=6.3) | “It is a digital health technological application called the ‘Sabi app’ which will be available to every individual in Nigeria through the use of their smartphones. The features of the Sabi app includes; the self-testing section and the e-counsellor platform.  Through the self-testing section, users will have access to HIV self-test kits which can be ordered and delivered to the user’s preferred location. Also, a DIY video tutorial will be available for users to learn how to use the HIV self-test kit. To improve supply and logistical issues of the self-test kit, we will form a strategic partnership with HI-TECH Diagnostic Limited (manufacturer of HIV self-testing kits in Nigeria) and a reputable inter-state delivery company in Nigeria.  The e-counsellor platform will enable users of the Sabi app to schedule an appointment with a medical doctor upon knowing his/her status using the self-test kit. The self-test kit delivered to the user will have a QR code in the delivery box. This will be scanned with the Sabi app in order to schedule a counseling session with a medical doctor. Thus, users not only know their HIV status privately but also seek further medical help privately too. This reduces stigmatization and empowers the users with sexual reproductive health information and services.” (#092, male, 24 yrs) |
| 3 - 3rd place  (Score=6.3) | “Although the ongoing COVID-19 pandemic has its laid down protocols which includes observing a 2 meter social distancing, wearing of nose mask, washing of hands with alcohol based hand sanitizer, avoiding hugs and handshakes. By putting these protocols in perspective, sensitizations and organizing of meetings and discussion sessions which may include visit to homes, schools mosques and churches which used to be the most effective medium as it involves physical explanations and heart to heart talks may no longer be adopted fully except in cases where the target audience is not large in number. To aid the sensitization process, religious leaders can be mobilized to help de-stigmatize the disease by preaching love and tolerance. They should encourage their followers to engage in regular check-ups, use prophylactics in situations where they cannot abstain or better flee from all forms of unsafe sex.  Secondly, during the pandemic, majority of the target audience spend most of their time on social media platforms, hence the virtual space can also be leveraged in sensitizing social media users in the Importance and uses of self-testing kits. This can be communicated via various social media platforms such as WhatsApp, Twitter, Facebook, etc., Information on what, how and when to use the self testing kits to carry out the self testing and to check the outcome. Short video kits can be produced and proliferated on social media platforms. These video kits would not exceed 2 – 5 minutes. Short descriptive articles can be written at least once a week explaining and describing all that is needed to be known about HIV and how to know and use the self testing kits. Also YouTube videos can be made on how to use the OraQuick HIV self-testing kit and the links would be sent using all available social media platforms in order to increase sensitization. Virtual meetings and group chat discussions can be organized and anchored where frequently asked questions can be attended to as well as solutions and recommendations can be proffered…Medical practitioners and/or consultants can be invited over the radio and television from time to time to host sessions on HIV, its issues, treatments and recommendations.  Also, information on self-testing can be conveyed via billboards, posters and flyers. Steps can be highlighted on how to use the self sampling kits, how to know their status, who to call and where to visit in a situation where the results turns out positive.  Furthermore, collaborations can be made with network service providers through the National Communication Commission (NCC) to send SMSs’ on “know your HIV status today” as well as a links underneath to refer them to sites where they can read up more on how to know their status as well as to use the self kits. Negotiations can be made with clinics and health centers to make these self testing kits readily available and relatively affordable. Also, free samples such as contraceptives and condoms can be shared to anyone who comes for further test after using the self testing kits as an incentive to encourage them to keep protecting themselves. Sensitization sessions can also be held at barbing salons as well as inscriptions about HIV can be posted on the walls to keep reminding intending customers on the to know their health status, who to talk to and where to visit for further instructions.” (#073, male, 24 yrs) |
