# Supplementary material for: Expanding Youth-Friendly HIV Self-Testing Services During the COVID-19 Pandemic: Qualitative Analysis of a Crowdsourcing Open Call in Nigeria
Source: JMIR Form Res. 2024 Apr 30;8:e46945. doi: 10.2196/46945 (PMC11094596; doi:10.2196/46945)
Supplement: Multimedia Appendix 2 [file formative_v8i1e46945_app2.docx]

| **Theme** | **Quotes** |
| --- | --- |
|  |  |
| Digital approaches (such as gamification, photo-verification system, and digital media) to generate demand for HIVST and avoid risks associated with attending clinics  (n=63) | “To make the idea of FISSK more exciting for young people, we design a FISSK mobile app with a very simple and friendly user interface. The FISSK app gives them step-by-step guidelines of how to use the FISSK to determine their immune strength. The attention should focus on the immune system and not the virus, and the relationship between the immune system and Covid-19. Also, the FISSK app is used as a tool that reminds users to regularly test for their immune strength. Also, over one million Nigerian youth use Twitter and Instagram and Facebook. We create the immune strength Self-test ads to promote the idea of self-testing on these social media platforms…The government and all concerned stakeholders interested in distributing Covid-19 Palliative should include the FISSK as part of the Covid-19 Palliative for youth as well as detailed instruction for usage while distributing the Palliative. Attention should be drawn to this: ‘No be Covid-19 Palliative go save you from Corona Virus. Na healthy immune system o’. Use the FSSK check am’.” (#016, male, 24 years) |
|  | “Another way to reach youth online is through their mobile. Apps can be created and offered to the public on HIV prevention, testing and counseling.  Social media is now a given in our daily lives. It is a big opportunity to promote HIV self-testing among youths especially during the ongoing COVID-19 pandemic. Online network or “social media” as we call it, allow people to link, interact, share and exchange information. Literally millions of people connect and share on social media every single day. But it is a great tool to promote HIV self-testing. Social media can be a platform that let you talk directly to the youth about HIV self-testing, share content, get involved in conversations, build trust, reach more people, grow your sphere of influence, and ultimately understand the youth better. Different social media network such as Facebook page, YouTube channel, Pinterest account, Twitter handles, Google+ page, LinkedIn company page can help promote HIV self-testing.” (#021, female, 19 years) |
|  | “For starters, nose-masks and hand sanitizers would remain vital commodities for as long as COVID-19 persists...Therefore, ViraCare is a double-pronged approach that proposes [1] an attractive and appealing product package of HIV Self-Testing kit alongside face-masks masks and hand sanitizers as incentives (ViraCare package) and [2] A mobile platform (ViraCare app) that provides (a) multi-lingual self-testing information and procedure for HIV and other STIs (b) online COVID-19 prevention and management information services (c) Seamless communication with medical experts for treatment, counselling and advice with option of anonymity, and lots more.” (#040, male, 20 years) |
|  | “The development of a mobile app is realistic as this will help manage the crowd that goes to hospital on daily basis for medical check-up, at the same time mitigate the spread of COVID 19.” (#045, female, 23 years) |
|  | “Our second tier is the use of a Multipurpose app which we someone can use to pay for stuff online and most importantly, Order Kits. The app has every information about the Organization. The reason for this app is to kill the stigma associated with going to the hospital to buy kits and to ensure confidentiality which one will not easily get in pharmacy stores. The reference number on each kit is linked to the app for easy tracking. After 24 hours of getting a kit, the app brings gives a pop up notification to ask if the individual has done the test. Another Notification comes up to ask the individual his/her status and also ask if the person wants to talk to a doctor if found positive. On this app also, we will have Online Challenges (Like the popular 10 years’ challenge), Games (Scrabble, Chess), Hotlines to Medical Counsellors for people that are positive to the virus. We plan to corroborate with the non-profit organization AHF (AIDS Healthcare Foundation) Nigeria that already provides free health services to HIV patients to link up our consumers with them and get them the medical counsellors or service they might need for free. The main aim for the app development is to reduce Human to human contact as COVID-19 vaccines have not yet been developed.” (#047, male, 21 years) |
| Awareness and sensitization through existing infrastructures (such as churches, schools, and health facilities)  (n=60) | “I also suggest that awareness should be given in other religious bodies, which I believe have youth groups and leaders. This way awareness is issued faster and conveniently. Due the pandemic and closure of tertiary institutions, is also a fast means of communication, since religious gatherings are accepted in the pandemic time.” (#018, female, 21 years) |
|  | “…so creating a forum for Educating the youth either through social media or even organising a seminar with strict covid-19 guidelines I believe I can be able to pass enough information to make them see reasons why knowing their status matters and why they need to know how to self-test.” (#028, female, 20 years) |
|  | “HIV self-testing through teaching or lecturing Lastly HIVST can be promoted in the on-going pandemic through teaching or lecturing, however there must be social distancing, use of facemask and adequate ventilation in the lecture room to facilitate understanding on how to use the seif-test kits at home or virtual learning can be introduced.” (#034, male, 18 years) |
|  | “HIV self-test outreaches and campaigns should be organized in university campuses, secondary schools and religious centers. Free test kits should be distributed during such events and cubicles should be made available for people to carry out the self test after being taught how to do it during the campaign. It is important to follow the COVID-19 guidelines during the outreaches in order to prevent the spread of the coronavirus.” (#072, male, 23 years) |
|  | “Although the ongoing COVID-19 pandemic has its laid down protocols which includes observing a 2 meter social distancing, wearing of nose mask, washing of hands with alcohol based hand sanitizer, avoiding hugs and handshakes. By putting these protocols in perspective, sensitizations and organizing of meetings and discussion sessions which may include visit to homes, schools mosques and churches which used to be the most effective medium as it involves physical explanations and heart to heart talks may no longer be adopted fully except in cases where the target audience is not large in number.” (#074, male, 24 years) |
| Partnerships with influencers, role models, and leaders (such as religious and youth leaders and social influencers in businesses, churches, organizations, and schools) to build trust in HIVST services  (n=56) | “If social media influencers are made special advocates and ‘direction givers’ for HIV self-testing kits, youths who are social media users will increase HIV testing as it still offers privacy and confidentiality. These influencers could offer special locations where the influencers could get their kits and testing services. They could also offer some form of direction for usage and candid advice after status check. These influencers will cut across local areas, special groups, professional bodies, media groups, art groups etc. As the effect of the COVID-19 pandemic is felt in Nigeria, these influencers would make it possible for people to acquire self-testing kits or access other HIV services without clustering or restriction to locations. This will be achieved as they will choose persons in their close areas to work with them. Some users will be able to access these kits for their peers and would advise them to do so in their convenience. They can share and talk about these in gatherings that the guidelines permit physically.” (#012, male, 21 years) |
|  | “Both entities (especially the mobile app) would be massively promoted via social media. I would indulge influential social media health advocates [Dr Chioma Nwakanma (Dr Zobo), Dr Fidelis Egemba (Aproko Doctor), Dr Olufunmilayo Harvey (Your Favourite Online Doctor) etc.] as well as local celebrities to endorse and promote the app. MyPaddi, a sexual health app, for instance got over 6000+ additional downloads on Playstore few days after popular Big Brother Naija Star, Tacha endorsed it via her Twitter handle.” (#040, male, 20 years) |
|  | “Important personnel like the Kings, Imam and pastors would also be sensitized so that they can as well reach out to the people. There would be a kind of collaboration with NYSC Health related CDS groups that can help promote the self-testing campaign. During the outreach, a short drama promoting self-testing can be displayed, and packages can be given which can as well be appealing to the youth to further promote the self testing campaign.” (#045, female, 23 years) |
|  | “Important personnel like the Kings, Imam and pastors would also be sensitized so that they can as well reach out to the people. There would be a kind of collaboration with NYSC Health related CDS groups that can help promote the self-testing campaign. During the outreach, a short drama promoting self-testing can be displayed, and packages can be given which can as well be appealing to the youth to further promote the self testing campaign.” (#045, female, 23 years) |
|  | “To start with, since self-testing is useful in covid-19 (i.e isolation), it could be efficient to carry out HIV self-testing during the isolation. The reason is because diagnosing people with HIV as early as possible after infection is better so that care and treatment would begin.  Influential stakeholders should get involved in order to assist financially or providing HIV self-test kits to youths to carry their tests on their own.” (#052, female, 15 years) |
